# SPTLC3 regulates plasma membrane sphingolipid composition to facilitate hepatic gluconeogenesis

**DOI:** 10.1016/j.celrep.2024.115054

**Published:** 2024-12-10

**Authors:** David Montefusco, Maryam Jamil, Daniel Canals, Siri Saligrama, Yang Yue, Jeremy Allegood, L. Ashley Cowart

**Affiliations:** 1 Department of Biochemistry, Virginia Commonwealth University, Richmond, VA 23298, USA; 2 Department of Medicine, Stony Brook University, Stony Brook, NY 11794, USA; 3 Lead contact

## Abstract

SPTLC3, an inducible subunit of the serine palmitoyltransferase (SPT) complex, causes production of alternative sphingoid bases, including a 16-carbon dihydrosphingosine, whose biological function is only beginning to emerge. High-fat feeding induced SPTLC3 in the liver, prompting us to produce a liver-specific knockout mouse line. Following high-fat feeding, knockout mice showed decreased fasting blood glucose, and knockout primary hepatocytes showed suppressed glucose production, a core function of hepatocytes. Stable isotope tracing revealed suppression of the gluconeogenic pathway, finding that SPTLC3 was required to maintain expression of key gluconeogenic genes via adenylate cyclase/cyclic AMP (cAMP)/cAMP response element binding protein (CREB) signaling. Additionally, by employing a combination of a recently developed lipidomics methodology, exogenous C14/C16 fatty acid treatment, and *in situ* adenylate cyclase activity, we implicated a functional interaction between sphingomyelin with a d16 backbone and adenylate cyclase at the plasma membrane. This work pinpoints a specific sphingolipid-protein functional interaction with broad implications for understanding sphingolipid signaling and metabolic disease.

## In brief

The key advances in Montefusco et al. demonstrate a novel function of the serine palmitoyl transferase subunit SPTLC3 in cyclic AMP-directed regulation of hepatic glucose production in the context of metabolic disease. It is shown that SPTLC3-derived sphingomyelin regulates adenylate cyclase activity at the plasma membrane.

## Graphical Abstract

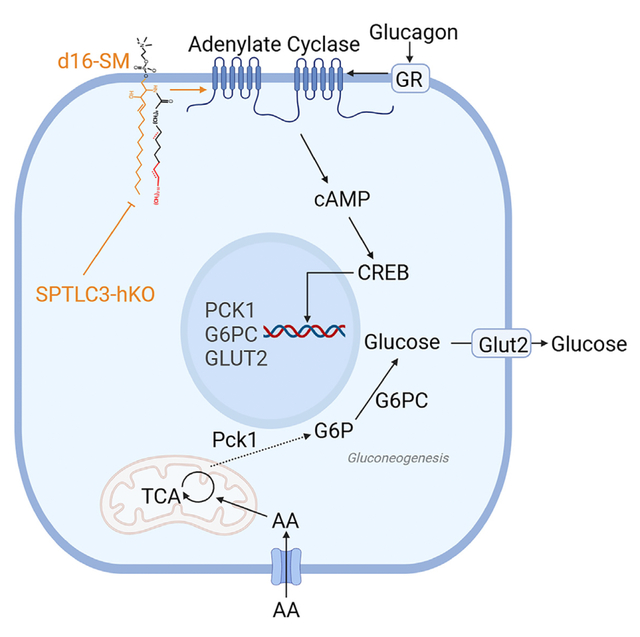

## INTRODUCTION

Sphingolipids have long been recognized as fundamental regulators of nutrient and energy metabolism that exert their effects through interaction with key proteins, including mammalian target of rapamycin (mTOR),^1,2^ protein kinase B (AKT),^3,4^ AMP-activated protein kinase (AMPK),^5–7^ peroxisome proliferator-activated receptors (PPARs),^7^ sterol regulatory binding proteins (SREBPs),^8,9^ cyclic AMP response element binding protein (CREB),^10,11^ and regulation of autophagy-related pathways.^12,13^ Sphingolipids have also been shown to mediate processes associated with diseases of overnutrition, such as non-alcoholic fatty liver disease (NAFLD) and type 2 diabetes (T2D).^14–18^

The first step of *de novo* sphingolipid biosynthesis is catalyzed by the serine palmitoyl transferase (SPT) complex, which uses serine and palmitoyl-coenzyme A (CoA) as substrates to produce an 18-carbon sphingoid base. The constitutive SPT complex is a multi-subunit complex most commonly consisting of two large subunits, SPTLC1 and SPTLC2, and one or more small subunits (ssSPTa/ssSPTb) and the orosomucoid-like proteins (ORMDL) regulatory subunits. By far the most abundant sphingolipids incorporate the 18-carbon dihydrosphingosine (DHS), of which the fatty chain includes the16 carbons from palmitate plus 2 carbons from serine.

Though d18-DHS-based sphingolipids constitute the major sphingolipid pools, some substrate promiscuity has been reported in SPT, largely associated with disease states, large changes in substrate abundance, and inherited point mutations in SPTLC2 and ssSPTa.^19–23^ However, substrate specificity is also substantially altered by substitution of the constitutively expressed SPTLC2 by its homolog, SPTLC3, which has low expression in most tissues. Inclusion of SPTLC3 in the SPT complex was initially reported by Hornemann et al. and has been shown to lead to production of a shorter 16-carbon sphingoid base, d16-DHS, derived from the 14-carbon myristoyl-CoA substrate and serine.^24,25^

The biological functions of SPTLC3 and its products in liver are not well understood. We have previously demonstrated a mechanistic link between high-fat diet, SPTLC3 upregulation in the heart, and increased d16-based sphingolipids, which exhibited proapoptotic effects in cultured primary cardiomyocytes.^25^ More recently, SPTLC3 has been found to be essential for function of the cardiomyocyte electron transport chain.^26^ However, most reports addressing SPTLC3 have been results from genomic screens evaluating the associations between SPTLC3 single-base genomic variants and circulating lipids and/or other disease markers.^27–44^ Despite the descriptive nature of these studies, some have provided tantalizing clues as to physiological functions of SPTLC3, especially in metabolic disease and its hepatic manifestation, non-alcoholic fatty liver disease (NAFLD). For example, multiple studies have reported that variants within and distal to the SPTLC3 gene had a strong relationship with hepatic lipid content,^42^ circulating lipids, and or lipoproteins.^33–36,38,43–46^ Importantly, other studies suggest a breadth of involvement of SPTLC3 in metabolic health, including T2D,^47^ insulin resistance,^36,38,41^ NAFLD,^42^ and cardiovascular disease.^33,44,46^ Despite these clues, there is no mechanistic information as to how SPTLC3 regulates metabolic processes. Here we report that d16-DHS derived from SPTLC3 is readily incorporated by ceramide synthases into a set of d16-based ceramides and a corresponding set of d16-based complex sphingolipids, including sphingomyelins, which concentrate in the plasma membrane and regulate glucagon-stimulated adenylate cyclase activity, thereby facilitating gluconeogenesis.

## RESULTS AND DISCUSSION

### Ablation of hepatocyte SPTLC3 prevents increased fasting blood glucose with high-fat feeding

SPTLC3 has been shown previously to be induced in the liver with a high-fat diet.^41,48^ Additionally, genome-wide association studies showed a relationship between SPTLC3 and NAFLD, T2D, and atherosclerosis.^40,49,50^ To investigate potential roles of SPTLC3 in NAFLD, we generated a conditional SPTLC3 knockout by introducing *loxP* sites flanking exon 8 of the SPTLC3 gene. Exon 8 contains the sequence for the pyridoxal 5-phosphate binding domain, which has been well established as the active site through studies on SPTLC2, which bears over 65% homology to SPTLC3.^23,51^ The conditional knockout was then crossed with albumin-cre (B6.Cg-Speer6-ps1^Tg(Alb-cre)21Mgn^/J) to generate an SPTLC3 hepatocyte-specific knockout (SPT3-hKO). Modification of the gene was virtually 100%, which was demonstrated by amplification of genomic DNA surrounding exon 8 (Figure S1).

**Figure 1.**
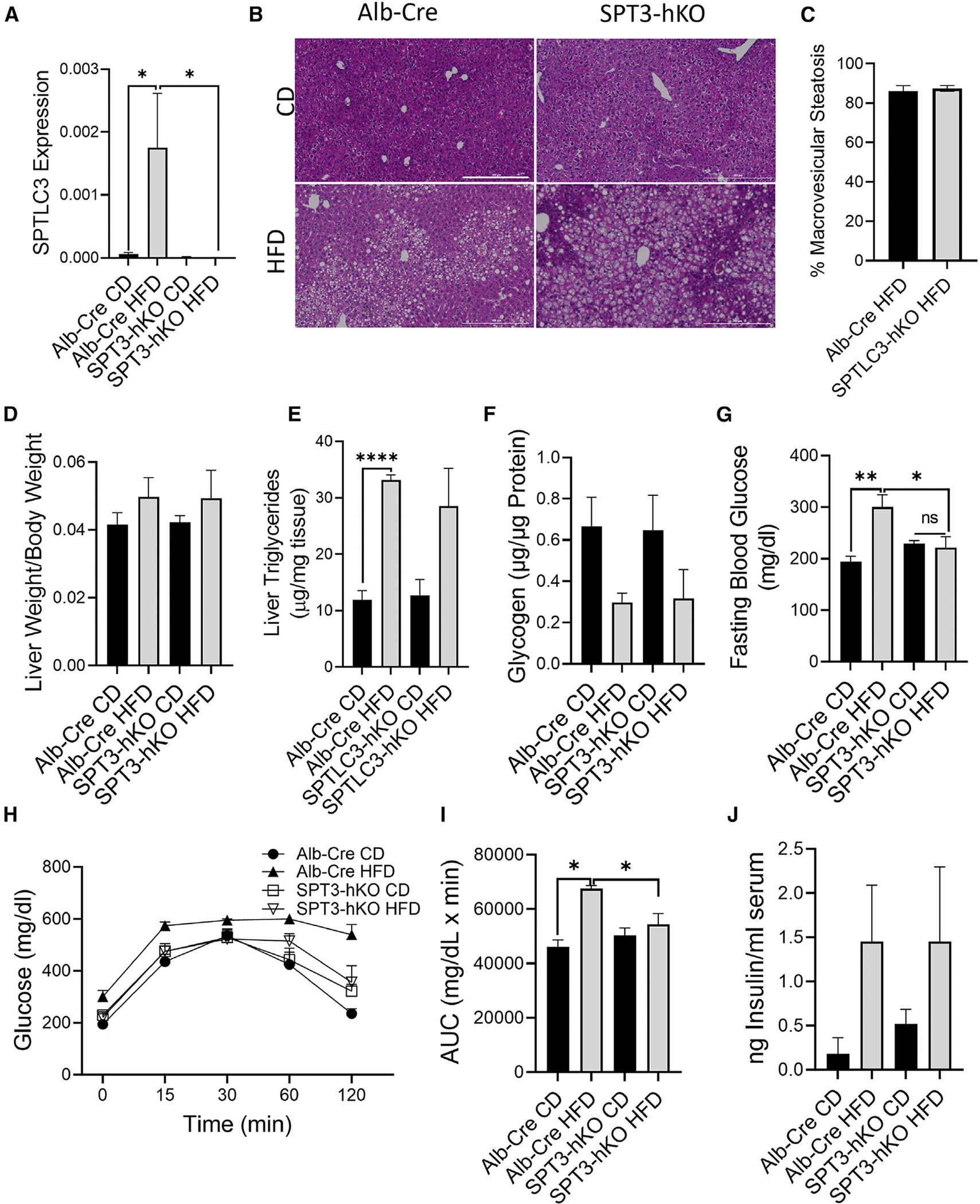
Male SPT3-hKO and Alb-Cre controls were placed on an HFD for 16 weeks and assessed for metabolic phenotype. (A) SPTLC3 expression in liver tissue homogenate of mice following high-fat diet (HFD) feeding versus low-glycemic control diet (CD) feeding. Expression was quantified by ΔΔcq method with a reference gene panel consisting of β-actin, Tbp1, and Hmbs1. (B) Representative images of H&E staining of liver tissue. Scale bars: 200 μm. (C) Quantification of macrosteatosis observed in histology images. (D) Liver weight. (E) TGs in liver homogenate. (F) Liver glycogen following HFD feeding. (G) Blood glucose following a 6 h fast. (H) ipGTT. (I) Area under the curve for the GTT. (J) Serum insulin. All graphical data are represented as mean ± SEM. The *p* values were calculated by Student’s t test. *p < 0.05, **p < 0.01, ****p < 0.001; *n* = 5 biological replicates.

The numerous observations of SPTLC3 being induced by high-fat feeding^41,48,50,52^ led to the hypothesis that SPTLC3 plays a role in the development of metabolic disease. Since the hepatic manifestation of metabolic disease is NAFLD, we hypothesized that SPT3-hKO may be protected from NAFLD phenotypes. To address this, the SPT3-hKO mice were subjected to high-fat feeding and assessed for NAFLD and/or T2D-related phenotypes. Liver tissue homogenate of male albumin-cre (Alb-Cre) control mice showed induction of SPTLC3 with high fat diet (HFD) feeding, while no expression was detected in SPT3-hKO (Figure 1A), confirming that the CRE-*loxP* system used to deplete SPTLC3 was effective. We assessed several measures of NAFLD progression, including steatosis, total triglycerides (TGs), fasting blood glucose, glucose tolerance, and hepatic glycogen. H&E-stained sections showed no apparent differences in steatosis with HFD feeding, which was confirmed by scoring for incidence of macrosteatosis (Figures 1B, 1C, and S3F), and there was no indication of difference hepatomegaly (Figures 1D and S3E). Consistent with these findings, there was also no difference in TG levels between Alb-Cre and SPT3-hKO mice (Figures 1E and S3D). Glycogen accumulation can also contribute to a steatotic phenotype, and glycogenolysis is the major source of hepatic glucose during short-term fasting; thus, liver glycogen levels were also measured, and no significant difference was observed (Figure 1F). Markers of inflammation, which increase in NAFLD, were also evaluated. Monocyte chemoattractant protein 1 significantly increased with HFD feeding, but there was no change in SPT3-hKO mice (Figure S2). Together, these data suggested that SPTLC3 is not a major player in HFD-induced NAFLD pathology.

To determine potential contributions of SPTLC3 to the overall HFD-induced T2D phenotype, an intraperitoneal glucose tolerance test (ipGTT) was performed following a 6-h fast. Prior to glucose injection, the Alb-Cre HFD group presented with elevated fasting blood glucose consistent with diet-induced metabolic disease (Figure 1G). In contrast, HFD-fed SPT3-hKO mice did not display elevated fasting blood glucose. Furthermore, after delivery of the glucose bolus, this difference persisted (Figures 1H and 1I). This suggested partial protection from the T2D phenotype, which may be analogous to reduced hepatic glucose production from antidiabetic drugs such as metformin.^53^ Though SPT3-hKO mice exhibited protection from HFD-induced elevated fasting glucose and impaired glucose clearance, HFD feeding elevated postprandial insulin similarly between genotypes (Figure 1J). Samples were taken postprandially due to experimental design. Suppression of elevated fasting blood glucose and differences in glucose tolerance were only observed in male mice (Figures S3A–S3C), which is not surprising since female mice are protected relative to male mice in the context of several diabetes models.^54,55^ While these data indicate that SPTLC3 may play some role in metabolic adaptations of HFD feeding, they also indicate that the attenuation of HFD-induced elevated fasting blood glucose arose outside of the context of general protection from NAFLD.

### Key regulators of gluconeogenesis are suppressed in SPT3-hKO primary hepatocytes

**Figure 2.**
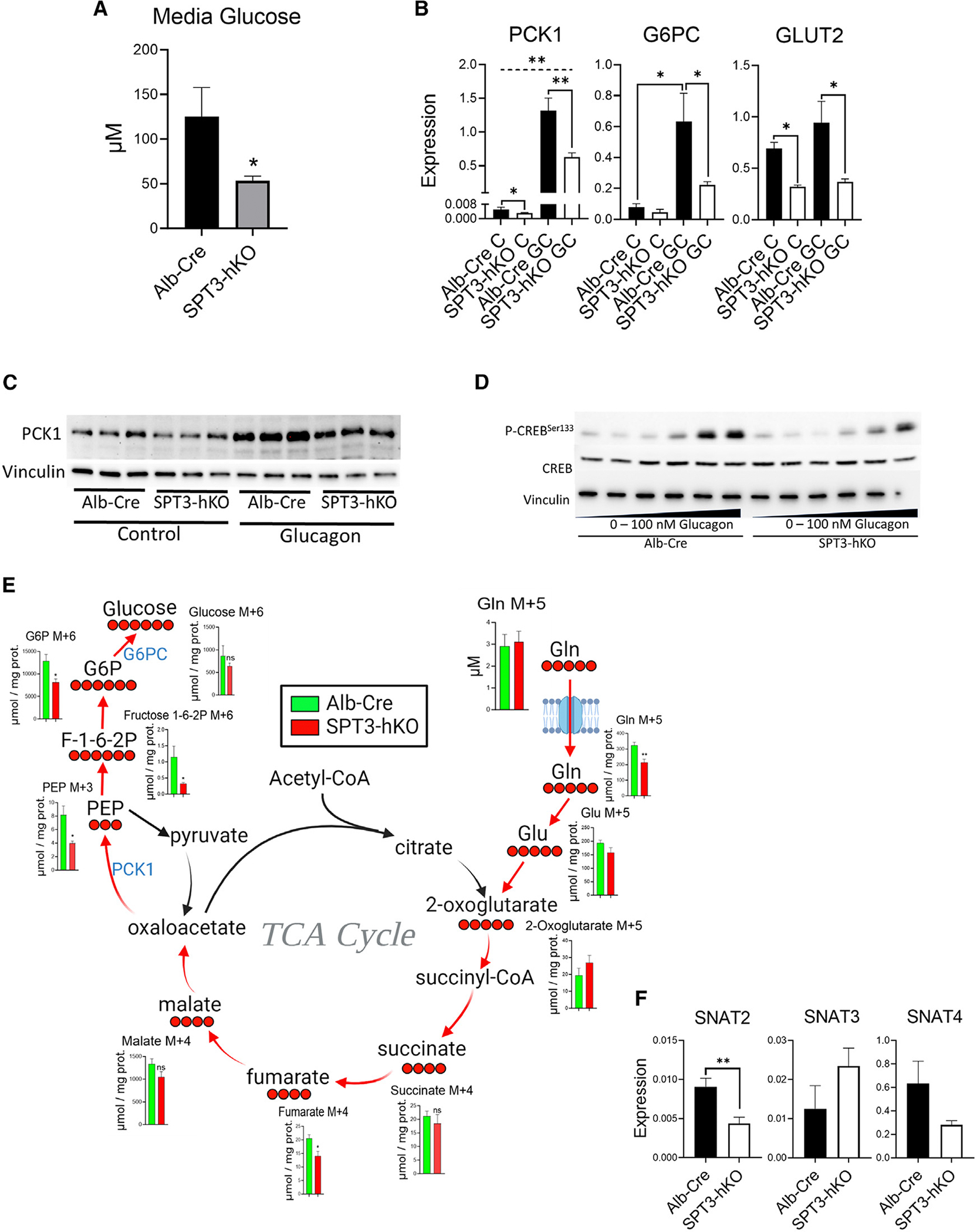
Evaluation of gluconeogenesis in primary mouse hepatocytes isolated from SPT3-hKO and Alb-Cre mice. (A) Glucose levels in medium produced by Alb-Cre and SPT3-hKO primary hepatocytes after 6 h. *p < 0.05 by t test. *n* = 5 biological replicates. (B) Expression by real-time qPCR of gluconeogenic genes following treatment with 100 nM glucagon (GC) for 3 h. Expression was measured by ΔΔcq method with a reference gene panel consisting of β-actin, Hmbs1, and Tbp1. Significance was determined by ANOVA. **p < 0.01, *p < 0.05. The dashed line indicates *p* < 0.01 for control versus GC for both Alb-Cre and SPT3-hKO. *n* = 3 biological replicates. (C) Western blot showing expression of PCK1 protein with GC treatment. This blot is representative of triplicate measurements. (D) Western blot showing P-CREB (Ser-133) phosphorylation in response to a GC dose of 0, 5, 10, 25, 50, or 100 nM. This blot is representative of triplicate measurements. (E) Stable isotopic labeling of metabolites feeding gluconeogenesis via the TCA cycle. Hepatocytes isolated from Alb-Cre and SPT3-hKO mice were treated with 4 mM U^13^C-glutamine for 6 h in DMEM without glucose, glutamine, or phenol red. Universally labeled target metabolites were targeted for analysis to assess the first pass through the TCA cycle. Cellular metabolite levels are expressed relative to total protein. The p values were calculated by t test. *p < 0.05, *n* = 6 biological replicates. (F) Expression of amino acid transporters in primary mouse hepatocytes by ΔΔcq method with a reference gene panel consisting of β-actin, Hmbs1, and Tbp1. Significance was determined by t test. **p < 0.01, *n* = 3 biological replicates. All graphical data are represented as mean ± SEM.

A critical function of the liver is to maintain blood glucose homeostasis during fasting through hepatic glucose production, which requires gluconeogenesis during long-term fasting. To recapitulate fasting *in vitro*, primary mouse hepatocytes isolated from Alb-Cre and SPT3-hKO were incubated for 6 h in glucose-free medium. During this time, Alb-Cre hepatocytes secreted enough glucose to accumulate to ~125 μM in medium, while SPT3-hKO produced only 50 μM, suggesting that glucose production may be impaired in SPT3-hKO hepatocytes (Figure 2A). Phosphoenolpyruvate carboxykinase (PCK1), which generates phosphoenolpyruvate, is the major rate-limiting enzyme in gluconeogenesis for substrates routed through the TCA cycle. Glucose-6-phosphatase (G6PC), which performs the final step in gluconeogenesis by dephosphorylating glucose-6-phosphate to release glucose, and GLUT2, which is the glucose channel through which hepatic glucose is exported, also play key roles in gluconeogenesis and/or maintenance of blood glucose levels during fasting, during which they are transcriptionally induced by glucagon signaling. To test whether induction of these genes was impaired in SPT3-hKO, we treated with 100 nM glucagon (GC) for 3 h. SPT3-hKO demonstrated very low basal expression of mRNA encoding these enzymes as compared to controls. Furthermore, while treatment with GC robustly induced these genes in control hepatocytes, message levels remained low in SPT3-hKO hepatocytes, suggesting impaired transcriptional induction (Figure 2B). Moreover, PCK1 protein increased notably with GC treatment in control cells but was markedly less in SPT3-hKO after GC (Figure 2C). PCK1, G6PC, and GLUT2 were not suppressed in post-prandial livers from diet mice (Figure S6); *in vivo*, it appears that onset of metabolic disease with fasting is required for an observable effect. CREB is among several signaling proteins that affect gene transcription in response to cyclic AMP (cAMP) levels. CREB is activated by phosphorylation at serine 133 in response to cAMP in response to GC. A GC dose response showed that P-CREB levels are suppressed in SPT3-hKO relative to Alb-Cre, approaching 100 nM (Figure 2D), implying that impaired cAMP signaling may be the cause of suppressed PCK1, G6PD, and GLUT2 expression.

This impaired PCK1 expression led to the hypothesis that blocked PEP synthesis was the major metabolic bottleneck leading to suppressed glucose production. However, sphingolipids have been implicated previously in several aspects of nutrient supply and metabolic regulation, such as nutrient uptake, mTOR regulation, starvation signaling, autophagy and others. By mass, the major gluconeogenic substrates *in vivo* are lactate and glutamine,^56^ and additional studies indicate that glutamine is a primary substrate for GC-mediated gluconeogenesis and that glutamine utilization is acutely sensitive to cAMP signaling.^57^ Untargeted metabolomics revealed that several metabolites that can act as gluconeogenic substrates were elevated in SPT3-hKO hepatocytes relative to controls, including lysine and glutamine (Figure S4), but not lactate. However, this analysis represented a “snapshot” of metabolism, and these elevations could indicate either increased flux through gluconeogenesis or a block in downstream steps of gluconeogenesis, leading to accumulation of gluconeogenic substrates. Thus, we chose to assess gluconeogenic activity by metabolite tracing. Primary hepatocytes from control and SPT3-hKO mice were treated with universally labeled ^13^C-labeled glutamine (U^13^C-gln) in glucose-free medium to track the exchange of carbons through entry into and exit from the TCA cycle and into the gluconeogenesis pathway.^56^ Universally ^13^C-labeled target metabolites were selected to isolate the first pass through the TCA cycle in the forward direction and the exit to gluconeogenesis through generation of PEP. Indeed, the distribution of labeled metabolites with respect to genotype was consistent with blocked PEP synthesis in the SPT3-hKO hepatocytes (Figure 2E). Specifically, M+3 PEP accumulated to about half the level in SPT3-hKO hepatocytes relative to Alb-Cre. This difference in PEP labeling was reflected in the downstream metabolites M+6 G6P and fructose 1,6-bisphosphate. While this was not reflected in the level of M+6-labeled intracellular glucose, it should be noted that the amount of labeled intracellular glucose M+6 is less than 10% that of G6P M+6, indicating that, as expected, more than 90% of the labeled glucose was exported but did not accumulate to detectable levels in the medium. However, the data in Figure 2A demonstrate significant reduction in glucose production by SPT3-hKO under the same culture conditions in glucose-free medium,.

Another finding from untargeted metabolomics was the decrease in intracellular Gln M+5 levels, which may imply an impairment of cellular glutamine uptake. Suppression of amino acid uptake is consistent with suppressed cAMP signaling; Ortiz et al. have shown that expression of the amino acid transporter SNAT2 is highly sensitive to adenylate cyclase activity and cAMP levels.^58^ We noted, in fact, that SNAT2 was basally suppressed in SPT3-hKO (Figure 2F), However, because the amount taken up was enough to drive conversion to 2-oxoglutarate to similar levels as in the wild type, we speculate that glutamine uptake under these conditions is not impaired sufficiently to compromise the kinetics of the downstream steps in the pathway independent from the decrease in PCK1 and therefore deemed this further evidence of impaired cAMP production, but overall, a less impactful effect than impairment in GC-stimulated PCK1. Thus, in total, these findings indicated that SPT3-hKO had impaired gluconeogenesis primarily due to suppressed conversion of oxalacetate to PEP and downstream metabolites.

### Hepatocyte SPTLC3 depletion impairs GC signaling by altering cAMP metabolism

**Figure 3.**
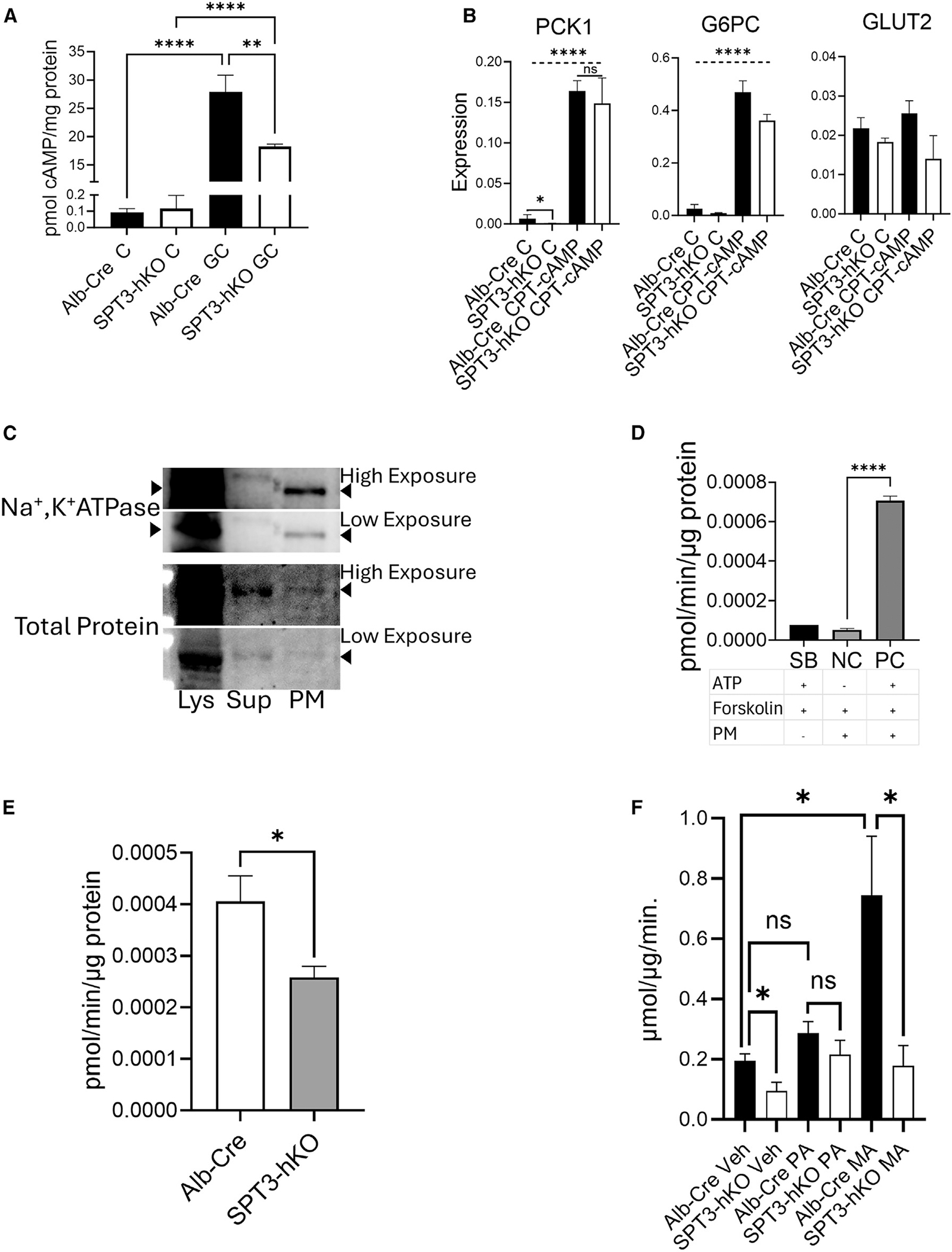
Evaluation of GC-driven cyclic AMP signaling in primary hepatocytes isolated from SPT3-hKO and Alb-Cre mice. (A) cAMP levels measured by ELISA following treatment of primary mouse hepatocytes with 100 nM GC. Significance was determined by t test. **p < 0.01, ****p < 0.0001. *n* = 3 biological replicates. (B) Expression by real-time qPCR of key gluconeogenic genes following treatment with cell-permeable cAMP chlorophenylthiol-cAMP (pCPT-cAMP). Expression was measured by ΔΔcq method with a reference gene panel consisting of β-actin, Hmbs1, and Tbp1. The dashed line indicates the level of significance between treated and control samples within each genotype. Significance was determined by ANOVA. *p < 0.05, ****p < 0.0001, *n* = 3 biological replicates. (C) Successful isolation of PM from whole liver was validated by western blot of sodium-potassium ATPase (Na^+^, K^+^ ATPase) in lysate (Lys), supernatant (Sup), and plasma membrane (PM) fractions. Replicate low- and high-exposure images capture the Lys and PM fraction bands, respectively. This blot is representative of the 5 replicate PM extractions used to generate AC activity data shown in (E). (D) *In vitro* AC activity was determined by measuring cAMP produced by partially purified PM extracts from whole liver. Positive control (PC) samples consisted of pooled PM extracts treated with an AC activator (24 μM forskolin). NC samples were prepared with PM extracts and no ATP substrate. Sample blanks (SBs) contained no PM extract. ****p < 0.0001; significance was determined by t test; *n* = 3 technical replicates. (E) AC activity of partially purified PM fractions from isolated livers of 5 individual HFD-fed mice. *p < 0.05; significance was determined by t test; *n* = 5 biological replicates. (F) *In situ* AC activity on hepatocytes isolated from Alb-Cre and SPT3-hKO littermate controls. Cells were pretreated for 24 h with 250 μM palmitate (PA), myristate (MA), or vehicle (0.25% DMSO in 2% fatty acid free BSA). Fatty acids were removed and replaced with 100 nM GC plus a phosphodiesterase/ATPase inhibitor cocktail. After a 30 min of pre-treatment with GC/inhibitors, 5 mM isotopically labeled ATP was added for an additional 30 min, and the reaction was quenched with 80% methanol. The isotopic cAMP product was quantified by LC-MS/MS, and specific activity was calculated using total protein. Significance was determined by individual t tests. **p < 0.01, *n* = 3 biological replicates. All graphical data are represented as mean ± SEM.

GC is the major driver of hepatic gluconeogenesis acting through GC receptors at the cell surface. GC receptors interact with adenylate cyclases (ACs), which also reside in the plasma membrane. ACs are responsible for synthesis of the major second messenger cAMP from ATP substrate and are the main regulators of cAMP levels. To test whether GC signaling was impaired in the SPT3-hKO, levels of cAMP were measured in Alb-Cre and SPT3-hKO cell lysates following treatment by GC (Figure 3A). As expected, GC increased cAMP levels in control hepatocytes; however, the cAMP increase was impaired in SPT3-hKO, which, together with the blunted increase of mRNA encoding gluconeogenic proteins and attenuation of GC-mediated CREB phosphorylation in SPT3-hKO cells, suggested that hepatocytes require SPTLC3 for normal GC stimulation of AC activity. It should be noted that PCK1 and GLUT2 expression was basally lower in untreated control cells, indicating that AC activity may be proportionally suppressed in SPT3-hKO relative to Alb-Cre regardless of input from the GC signaling pathway. Because the first step in GC signaling is activation of cAMP production, we tested whether exogenous cAMP could bypass the putative GC signaling defect in SPT3-hKO hepatocytes. To this end, cells were treated with chlorophenylthiol (CPT)-cAMP, a cell-permeable cAMP derivative (Figure 3B). CPT-cAMP increased mRNA expression of PCK1, G6PC, and GLUT2 to a similar extent in SPT3-hKO and Alb-Cre, implying that the defect in GC signaling mediated by SPTLC3 may arise from impaired cAMP production at the plasma membrane.

Because SPTLC3 plays a role in biosynthesis of sphingolipids, which are essential membrane components, we speculated that an SPTLC3-derived sphingolipid may be required for production of cAMP at the plasma membrane. This idea is consistent with previous studies demonstrating that AC activity is sensitive to its membrane environment,^59–62^ which, together with data thus far, led to the hypothesis that SPTLC3-derived sphingolipid products may contribute to the plasma membrane environment in a manner that facilitates AC activity. DHS, which is the first readily detectable product arising from the SPT complex, is a substrate for a family of ceramide synthases, which utilize acyl-CoA substates of between 14 and 26 carbons to generate dihydroceramides. Desaturation of these dihydroceramides generates a corresponding set of ceramides, which can then be incorporated into more complex sphingolipids, including sphingomyelin, which is abundant in the outer leaflet of the plasma membrane. Therefore, d16-DHS, derived from SPTLC3, could become incorporated into dihydroceramides, ceramides, and sphingomyelins with d16-sphingoid backbones, which may impact cell membrane properties, thereby affecting AC activity. To address this hypothesis, partially purified plasma membrane fractions were isolated from steatotic Alb-Cre and SPT3-hKO mouse livers.^63^ The plasma membrane marker sodium-potassium ATPase (Na^+^, K^+^ ATPase) was probed by western blot to demonstrate successful enrichment of the plasma membrane (Figure 3C). Endogenous AC activity was measured in these purified fractions by quantifying the production of cAMP following addition of ATP substrate.^64^ The positive control (PC), negative control (NC), and sample blanks (SBs) contained a combination of ATP substrate, plasma membrane (PM) fraction and/or AC activator forskolin (Figure 3D). Indeed, PM fractions obtained from the liver homogenates of HFD-fed mice were assayed, and the forskolin-induced production of cAMP from ATP was reduced by approximately 30% in SPT3-hKO relative to control hepatocytes, indicating impaired PM AC activity (Figure 3E). To further establish a dependence of cAMP production on SPTLC3, *in situ* cAMP activity was measured in Alb-Cre and SPT3-hKO hepatocytes. Cells were first pretreated with palmitate or myristate for 24 h to trigger an increase in d18.1 and d16.1 sphingolipids, respectively. As predicted, the level of AC activity was basally suppressed in SPT3-hKO treated with vehicle. Interestingly, myristate (MA) pretreatment led to the greatest increase in activity in Alb-Cre, with no significant increase in MA-treated SPT3-hKO. While palmitate (PA)-pretreated cells showed no changes in AC activity and no significant difference between genotypes (Figure 3F). Together, these data imply that SPTLC3-derived d16-based sphingolipids are key regulators of AC activity.

### SPTLC3 generates d16-based PM sphingomyelins

AC activation requires dimerization, which is sensitive to the membrane environment.^65,66^ Sphingomyelin is a major component of the PM in many cell types, including hepatocytes, and has been linked to glucose metabolism in hepatocytes^66,67^ and other cell types^67^ and to nutrient and energy metabolism more generally.^68^ Sphingomyelins have also been associated with glucose metabolism in the context of metabolic disease.^15,69,70^ Therefore, we sought to assess SPTLC3-derived d16-based sphingomyelins.

**Figure 4.**
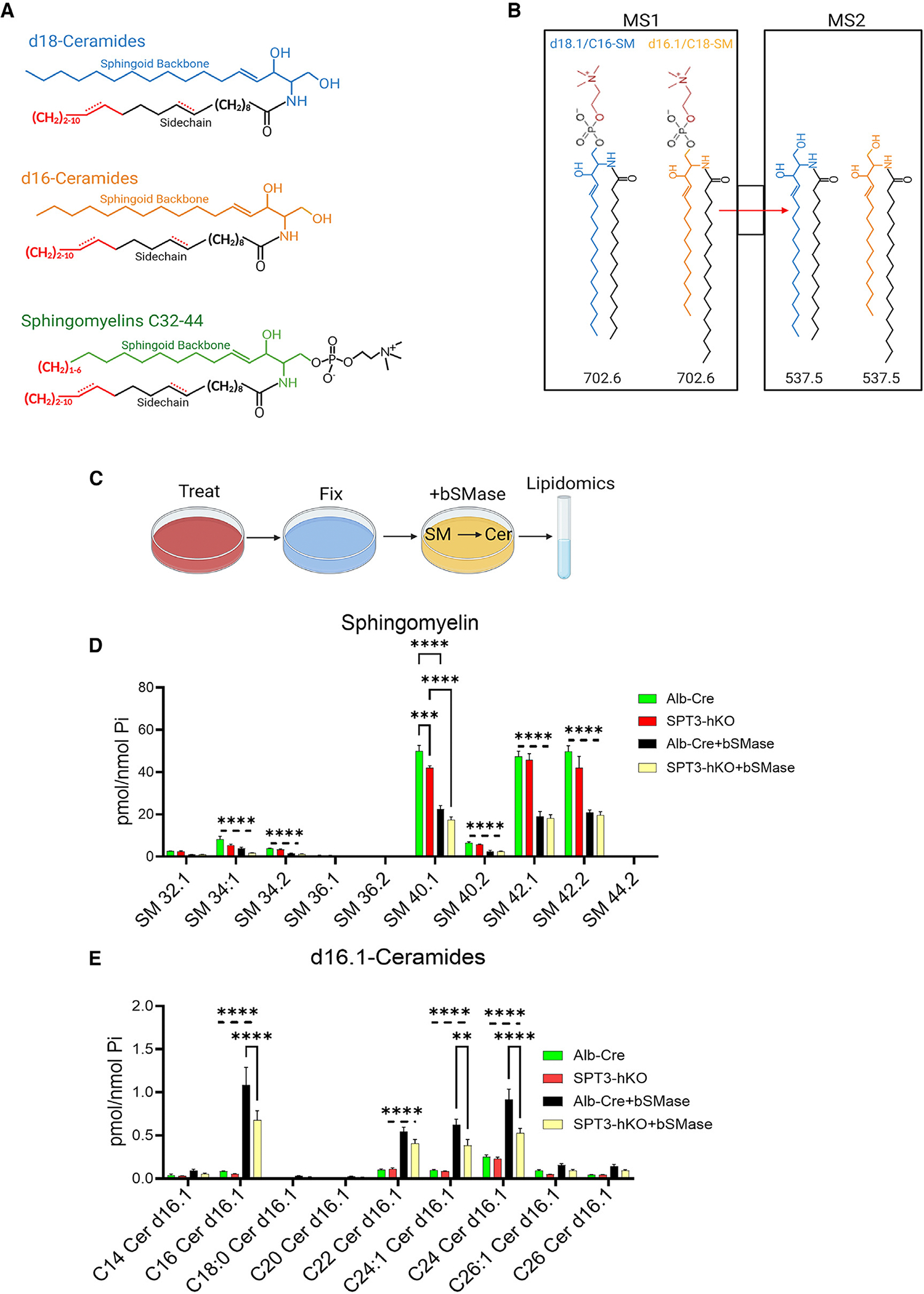
Lipidomics analysis revealing sphingoid base composition of PM sphingomyelin pools in primary hepatocytes isolated from SPT3-hKO and Alb-Cre mice. (A) Structures of ceramides and sphingomyelin, highlighting the range of reported side chains incorporated into the d16 or d18 sphingoid backbone. Current methods for quantification of sphingomyelins do not distinguish between side chain versus backbone. (B) Schematic of the MS/MS transition used to identify sphingomyelin peaks, illustrating the limitations of standard methods for determining backbone and side-chain composition. All illustrations were produced with BioRender. (C) Schematic of bacterial sphingomyelinase (bSMase) post treatment, which allows the identification of the backbone and side-chain structures of PM sphingomyelin in primary mouse hepatocytes. (D) Total sphingomyelin levels determined by LC-MS/MS on Alb-Cre and SPT3-hKO primary mouse hepatocytes with control or bSMase post treatment. Total cellular SM is shown in Alb-Cre and SPT3-hKO without post treatment. Alb-Cre+bSMase and SPT3-hKO+bSMase reveal the amount of SM after PM SM has been converted to ceramide. The difference between the two groups reveals the initial level of PM SM. Individual SM species are labeled by total carbons and double bonds. (E) D16.1-based ceramide levels determined by LC-MS/MS on Alb-Cre and SPT3-hKO primary mouse hepatocytes with control or bSMase post treatment. The *p* values were determined by ANOVA. **p < 0.05, ****p < 0.0001. *n* = 5 biological replicates. All data are represented as mean ± SEM.

While the backbone and side-chain composition of ceramides can easily be distinguished by liquid chromatography-tandem mass spectrometry (LC-MS/MS) methods (Figure 4A), this is less straightforward for sphingomyelins due to the diagnostic fragmentation pattern, which for SM relies on loss of the choline head group, leaving the intact ceramide as the product ion (Figure 4B). Though in the past it has often been assumed that the sphingoid backbone of sphingomyelin is 18 carbons, in practice it is difficult to pinpoint the backbone and side-chain length of specific SM regioisomers by LC-MS/MS. For each LC-MS/MS sphingomyelin peak, it is only possible to report the number of carbons and double bonds of the tail group fragment with confidence. This issue is discussed further in the STAR Methods.

To circumvent the challenges of accurate assignment of sphingoid base composition of sphingomyelins and to specifically target PM sphingomyelin, the method of Greene et al. was employed.^71^ In brief, cells were fixed and then treated with bacterial sphingomyelinase (bSMase), which hydrolyzes cell-surface sphingomyelins on the outer leaflet of the PM (Figure 4C). The bSMase treatment converts all outer-leaflet SM to ceramide. The liberated ceramide can then be measured using ceramide-specific LC-MS/MS methods, whereby the backbone/side-chain fragmentation pattern allows the backbone composition of the liberated ceramide to be readily identified. Prior to bSMase, the SM-specific LC-MS/MS method was only able to reveal that 40.1-SM showed a significant decrease in SPT3-hKO (Figure 4D). This SM species, identified as a peak in the LC-MS/MS chromatogram, likely arises largely from the sum of SM-d18.1/22 and SM-d16/24.1. However, bSMase treatment revealed that depletion of SPTLC3 reduced PM levels of d16.1/C16, d16.1/C24.1, and d16.1/C24 sphingomyelins (Figure 4E). While d18.1-ceramides were also decreased in SPT3-hKO (Figure S5), these data suggest that PM d16.1/C16, d16.1/C24.1, and d16.1/C24 sphingomyelins comprise the major SPTLC3 products and, thus, are likely to be major contributors to the PM lipid microenvironment required for GC-stimulated AC activity.

### Limitations of the study

While these studies support the idea that SPTLC3 induction in HFD-induced obesity plays a role in elevated blood glucose in our mouse model, the relevance of this to humans is limited. However, several genome-wide association studies (GWASs) hint at a link between SPTLC3 and metabolic disease in patients as well as a role for SPTLC3 in supplying circulating sphingomyelins and ceramides. SPTLC3 has also been shown to be involved in the progression of patients from NAFLD to hepatocellular carcinoma.^50^ Furthermore, while we have linked alterations in PM sphingomyelin composition to AC function, it is likely that numerous membrane proteins require a precise membrane composition for homeostatic function. Therefore, the suitability of SPTLC3 as a therapeutic target to decrease GC-stimulated gluconeogenesis remains in question. However, though SPTLC1 and STPLC2, which comprise the constitutive SPT catalytic complex, are essential in mammals, constitutive SPTLC3 expression is low in most tissues, and, therefore, SPTLC3 may serve as an attractive target.^23^

While we demonstrated a correlation between altered PM sphingolipids and impaired AC activity, further molecular mechanistic details of how SPTLC3-generated sphingomyelins may interact directly with AC remain to be determined. Supporting a role for direct lipid-protein interaction for AC activity, mycobacterial Rv2212 AC is strongly activated by fatty acids,^72^ and the crystal structure of the closely related Rv1264 revealed a bound oleic acid.^73^ These bacterial enzymes are closely related to the mammalian class III ACs and support the possibility of a hydrophobic pocket in AC capable of binding the sphingoid base or side chain of a sphingomyelin molecule.

The mechanism of SPTLC3 induction during HFD feeding is still unknown. Single-base variants identified in humans that correlate to metabolic phenotypes occur distal to the SPTLC3 gene near putative transcription factor binding sites that regulate glucose metabolism. Specifically, SNPs rs364585 and rs6109681 are both linked to metabolic disease and are adjacent to a putative HNF4 binding site (Motifmap; https://motifmap.ics.uci.edu/). The mechanisms of SPTLC3 regulation and function of downstream sphingolipids under numerous pathological conditions are the subjects of current studies.

## RESOURCE AVAILABILITY

### Lead contact

Requests for further information, resources, and reagents should be directed to and will be fulfilled by the lead contact, L. Ashley Cowart (lauren.cowart@vcuhealth.org).

### Materials availability

This study did not generate any unique reagents.

### Data and code availability

- The accession number for the raw untargeted metabolomics data can be found in the key resources table.
- No new code was generated for this manuscript.
- Any additional information required to reanalyze the data reported in this paper is available from the lead contact upon request.

## STAR★METHODS

Detailed methods are provided in the online version of this paper and include the following:

### EXPERIMENTAL MODEL AND STUDY PARTICIPANT DETAILS

#### Generation of the SPT3-hKO mouse

The ‘floxed’ conditional knockout mouse was crossed with Albumin-Cre (B6.Cg-Speer6-ps1Tg(Alb-Cre)21Mgn/J) obtained from Jackson Laboratories. Successful excision was confirmed by amplification of the targeted region using two primers outside of the excised region, and a third within exon 8. This primer set resulted in a 616bp amplicon for the wild type gene, and a 343bp fragment following excision by the cre recombinase (Figure S1). The primer sequences are given in Figure S1. Once established, the colony was backcrossed with fresh male and then female albumin-cre mice obtained from Jackson Laboratory.

#### Approval of animal studies

All animal experiments conformed to the National Institutes of Health *Guide for the Care and Use of Laboratory Animals* and were in accordance with Public Health Service/National Institutes of Health guidelines for laboratory animal usage.

#### High fat diet

The experimental groups consisted of male and female Albumin-Cre and SPT3-hKO C57BL/6 mice. Mice were group-housed in the animal facility at Virginia Commonwealth University. Food and water were provided *ad libitum*, animals were maintained on a 12:12 h light-dark cycle and ambient temperature was steadily 21°C. Littermate controls were randomized to a high saturated fat diet (HFD) (Envigo, TD.09766) (60% kcal provided by milkfat) or an isocaloric low-fat diet (CD) (Envigo, TD.120455) (17% kcal provided by lard) at 10 weeks of age, and diets were administered for 16 weeks (*n* = 5 per group). Mice were euthanized humanely by isoflurane (Hospira, Inc., Lake Forest, IL) followed by cardiac puncture. Cardiac blood was prepared for non-hemolyzed serum, aliquoted, and stored at −80°C. Tissues were collected accordingly as fresh fixed in 10% neutral buffered formalin or fresh snap-frozen in liquid nitrogen and stored at −80°C. All study methods were approved by the IACUC board of Virginia Commonwealth University.

### METHOD DETAILS

#### Intraperitoneal glucose tolerance test

Mice were fasted for 6 h starting at 8 in the morning, and fasting blood glucose was measured prior to administration of glucose. Glucose was administered by intraperitoneal injection as a 20% glucose solution in saline with a volume determined based on 2 g of glucose/kg body mass. Blood was collected through a nick in the tip of the tail after disinfecting with 70% ethanol.

#### Liver triglyceride levels

A 50 mg tissue sample was dissolved in 6x volume of 2:1 ethanol/30% KOH at 60°C for 5 h, with periodic vortexing to improve digestion. A volume of 1.08 × the original volume of 1 M MgCl_2_ was added, vortexed, and chilled on ice for 10 min. The digest was centrifuged at 14 000 × g for 30 min at room temperature, and supernatant was collected and diluted 1:10 in water for use in the triglyceride assay. The supernatant was measured with the Triglyceride LiquiColor Test (Stanbio 2200-225).

#### Liver glycogen

A 50 mg section of tissue was homogenized in 1 mL of ddH_2_O, with 10–15 passes in a Dounce homogenizer. Homogenate was boiled for 10 min, centrifuged at 4°C, 10 min, 18 000 g. Supernatant was transferred to a new tube, and an aliquot was taken for BCA. Glycogen was quantified by the Abcam Assay Kit (ab65620).

#### Serum insulin

Blood was harvested by cardiac puncture under isoflurane anesthesia. Non-hemolyzed insulin was quantified by the mouse insulin ELISA kit from Crystal Chem (90080).

#### Isolation of primary mouse hepatocytes

Isolation was carried out based on the method of Poggel et al.^74^ Briefly, the inferior vena cava (IVC) was catheterized under isoflurane anesthesia, the IVC was clamped anterior to the heart, and then the liver was flushed from the portal vein with 3 mL of heparin saline, 25 mL of perfusate 1 containing EGTA, and then perfusate 2 containing collagenase. Hepatocytes were isolated by Percoll gradient and cultured in William’s E media (Gibco) with 10% FBS, and supplemented with insulin, dexamethasone, and pen/strep. Cells were plated on collagen coated dishes (Collagen solution, Cat. No. C8919), at 11–12 in the afternoon, and then media was exchanged 4 h after plating. Experiments were initiated the following morning at 8–10 a.m.

#### Hepatic glucose production

Primary hepatocytes isolated from Alb-Cre and SPT3-hKO littermate control mice were cultured normally in supplemented William’s E media. In the morning following harvest cells were serum starved for 3 h in DMEM after which media was aspirated, and cells were rinsed twice with DMEM containing no glucose, serum nor phenol after which this media was left on for 8 h. Glucose was quantified in the media by LC-MS/MS.

#### Cell treatments

Experiments on primary mouse hepatocytes were carried out in DMEM (Gibco,10-013-cv) with 10% FBS, and initiated at 8–10 the morning following isolation. Cells were treated with glucagon (Millipore/Sigma G2044), or pCPT-cAMP (Sigma C3912, 8-(4-Chlorophenylthio) adenosine 3′,5′-cyclic monophosphate sodium salt); all treatments were soluble in media with no need for additional vehicle.

#### Gene expression by qPCR

Cells were scraped from a 6-well dish into 0.2 mL Trizol, liver tissue was homogenized in 1 mL of Trizol per 20 mg of tissue. Tissue was homogenized by magnetic bead homogenizer at the maximum power setting for 3× 3min cycles, resting in an ice bath for 1 min between cycles. RNA was extracted by addition of 40 μL or 200 μL of chloroform for cells and tissue respectively. Sample mixtures were shaken 20 times by hand and then allowed to rest at room temperature for 3 min, and then centrifuged at 4°C for 10 min at 12 000 × g. The top layer containing extracted RNA was removed and cleaned up using the Qiagen RNAeasy kit, and then reverse transcribed using the Biorad iScript kit. A Sybr green protocol with a 58°C annealing temperature for 40 cycles was used with primers listed in the key resources table. Mean normalized expression was calculated relative to a reference gene panel consisting of βActin, TBP1 and HMBS1 using the ΔΔcq method.

#### Western blot

RIPA buffer containing Pierce protease and phosphatase inhibitors was added to liver tissues (50 mg/mL) or primary mouse hepatocytes (0.2 mL per well). Liver samples were homogenized with a magnetic bead homogenizer at the maximum power setting for 3× 3min cycles, resting in an ice bath for 1 min between cycles. Cell or tissue proteins were quantified by BCA and prepared in 4x Laemelli buffer at 1 mg/mL, and 15 μg was loaded per well of a 26-lane 4–15% BioRad Criterion gradient gel. Nitrocellulose membranes were blocked and then blotted in TBST with 5% milk or 5% BSA according to manufacturer’s recommended protocol at 1:1000 dilution. The following primary antibodies were acquired from Cell Signaling Technology: P-AKT (9271), P-AMPKα (#2535), PCK1 (D12F5, #12940), P-CREB (#9198). Anti-Rabbit mAb 12940 secondary antibody was used for all primaries at a 1:5000 dilution in TBST with 5% BSA.

#### Cyclic-AMP measurements

Cyclic AMP (cAMP) was measured in primary mouse hepatocytes by ELISA. Media was aspirated and 1mL of 0.1 M HCl was added per well, and then incubated 20 min at RT, scraped and collected, homogenized by pipet, and then centrifuged 1000 × g for 10min. Supernatants (750 μL) were collected, 1 mL of ELISA buffer was added, an aliquot was removed for quantification of protein by BCA, and then samples were stored at −80°C. Measurements were performed with an ELISA kit (Cayman 581001).

#### Plasma membrane isolation

Partially purified plasma membrane extracts were generated based on Suski et al.^63^ A set of 10, 100 mg liver sections from Alb-Cre and SPT3-hKO HFD mice were washed twice with 1 mL ice-cold starting buffer (250 mM mannitol, 75 mM sucrose, 30 mM Tris-HCl, pH 7.4), and then homogenized with 8–10 strokes in a manual Dounce homogenizer in 400 μL of isolation buffer (starting buffer plus 0.5% BSA and 0.5 mM EGTA). Homogenates were centrifuged at 800 × g for 5 min, the supernatant was collected in spun again at 800 × g for 5 min, and then an aliquot was collected and labeled ‘lysate’ for validation by Western blot. The supernatants were centrifuged at 10 000 × g for 10 min, the supernatant was collected in spun again at 10 000 × g for 10 min. Supernatants were then centrifuged at 25 000 × g for 20 min, resuspended in 400 μL of starting buffer, and then centrifuged again at 25 000 × g for 20 min. The supernatant was collected ‘supernatant’ sample for validation by Western blot The pellet was resuspended in 200 μL of Buffer A (100 mM Tris), and an aliquot was collected for ‘plasma membrane’ sample for validation by Western blot. Total protein was measured by BCA assay, and protein concentrations were equalized by dilution with Buffer A.

#### *In vitro* adenylate cyclase activity

Adenylyl cyclase activity in plasma membrane extracts was measured based on Marchmont et al.^62^ Cyclic-AMP was generated from ATP substrate in plasma membrane extracts with equalized protein concentration. The reaction buffer consisted of 2 mM MgCl_2_, 0.5 mM NaF, 2 mM pan-phosphodiesterase inhibitor 3-Isobutyl-1-methylxanthine (IBMX). Reactions were initiated by addition of 5 mM ATP, incubated at 37°C for 30 min, and then quenched by boiling for 1 min. Positive control, negative control, and sample blank (PC, NC, SB) were first prepared to measure the level of background and establish the dynamic range of the assay. The sample blank contained ATP substrate with no PM fraction, the NC contained PM fraction (pooled from all liver samples) with no substrate, and the PC contained the PM fraction, ATP, and the AC activator forskolin (Figure 3D). Positive and negative control samples were prepared using pooled plasma membrane extracts with 24 μM Forsklin with or without ATP substrate respectively. Sample blanks were prepared with reaction buffer components and ATP without PM extracts. Protein was precipitated in methanol and cAMP was quantified by LC-MS/MS.

#### *In situ* adenylate cyclase activity

Albumin-cre and SPTLC3-hKO littermate control mice were selected for primary hepatocyte isolation on day 0 of the treatment course. On day 1 cells were treated with 250 μM palmitate, myristate or the equivalent vehicle volume. Fatty acid stocks were prepared by conjugating to fatty acid free BSA. Fatty acid or vehicle (0.25% DMSO) were added to a 2% BSA solution and then sonicated in a bath sonicator for 15 min at 45°C. After 24 h of fatty acid treatment, fatty acid media was replaced with fresh media and inhibitors plus glucagon were added. Inhibitors were added to maintain the pool of fresh cAMP to ensure that the level of isotopically labeled cAMP was reflective of adenylate cyclase activity. The inhibitor cocktail consisted of the phosphodiesterase inhibitor 3-Isobutyl-1-methylxanthine (IBMX), and ATPase inhibitors digoxin and thapsigargin. Glucagon was delivered at 100 nM and inhibitors were delivered at 2 mM, 2 μM, and 200 nM respectively. Following a 30 min pre-treatment with glucagon plus inhibitors, 5 mM isotopically labeled ATP (adenosine-^13^C10,^15^N5, 5′-Triphosphate) was added for 30 min. The reaction was quenched by adding 80% methanol. Cells were scraped into 80% methanol and protein precipitate was collected for normalization by total protein. Isotopically labeled cAMP was quantified by LC-MS/MS.

#### Quantification of labeled cAMP by LC-MS/MS

*Chemicals and reagents*: Isotope labeling of cyclic AMP internal standard (HY-B1511S) and ATP (Cat 645702) were purchased from MedChemExpress (Monmouth Junction, NJ) and Sigma Aldrich (St. Louis, MO) with the minimal 95% purity or the highest available purity. Solvents used for metabolites extraction and liquid chromatography including hexafluora-2-isopropanol (HFLP), triethylamine (TEA), formic acid, acetonitrile, methanol and water were LC-MS grade from Fisher Scientific (Minneapolis, MN). *Sample Treatment.* A total of 450 μL cold methanol was added into 150 μL reaction solution, the protein was removed after centrifuge at 4°C for 10 min at 13000 rpm. The supernatant was dried in speed-vac concentrator (SPD 2010 Savant, Thermo) and reconstituted with 100 μL H2O. 5 μL was injected into the LC-MS/MS system. *LC-MS/MS Measurement.* The analysis of cyclic AMP, AMP, ADP and ATP were operated on an AB SCIEX (Foster City, CA) QTRAP 6500 system, which consists of an SHIMADZU Nexera ultra high-performance liquid chromatography system coupled with a hybrid triple quadrupole and ion trap mass spectrometer. Analyst 1.6 software was used for system control and data acquisition, and MultiQuant 3.0 software was used for data processing and quantitation. Cyclic AMP was separated with reverse-phase LC with Thermo Accucore Vanquish C18+ column (2.1 mm × 150 mm, 1.5μm). The Isocratic method was used with the mobile phase of 0.2% formic acid in water: acetonitrile (80 : 20) at the flow rate of 0.13 mL/min at 35°C for 6 min. Ion-paring method was applied to separate AMP, ADP and ATP with an Atlantis T3 column (2.1 mm × 100 mm, 3.0μm) at the flow rate of 0.5 mL/min at 30°C. 100 mM HFIP and 8.6 mM TEA in water (final pH, 8.3 ± 0.1) was optimized as mobile phase A, and 10% acetonitrile in mobile phase A was used as mobile phase B. LC gradients starts at 0% B for 0.5 min, reached 10% B at 8 min, ramped to 100% B at 13 min, hold for 1 min and dropped to initial 0% B at 14.1 min. A quality control of 1 μM standard was used to monitor the intensity variation of every 10 injections of samples. The mass spectrometric parameters of ionization polarity, product ion, collision energy (CE), decluttering potential (DP) and cell exit potential (CXP) of all metabolites were manually tuned and optimized for best sensitivity by direct infusion (below table). Multiple reaction monitoring (MRM) mode with the above optimal parameter were used for analysis with Ion Spray (IS) potential of 4500 V for negative mode with nebulizer gas (GS1) and bath gas (GS2) at 40 psi and 25 psi respectively. Curtain gas (CUR) was 30 psi, and collision gas (CAD) was set to medium level; source temperature (TEM) was 450°C. The dwell time for each transition was optimized ranging from 150 to 200 ms.^75^

**Table:** 

MS/MS transitions and instrument parameters for key analytes					

Metabolite	Quadrupole 1	Quadrupole 3	Declustering Potential	Collision Energy	Collision Cell Exit Potential

cAMP	328.1	134.1	−80	−37	−10
Isotope cAMP from^13^C_10_,^15^N_5_ ATP	343.1	144.1	−80	−37	−10
^13^C5 cAMP internal standard	333.1	134.1	−80	−37	−10
ATP	506.1	407.8	−34	−32	−15
^13^C_10_,^15^N_5_ ATP	521.1	422.8	−34	−32	−15
ADP	426	327.8	−65	−24	−22
Isotope ADP from^13^C_10_,^15^N_5_ ATP	441	342.8	−65	−24	−22
AMP	346	78.8	−130	−70	−10
Isotope AMP from^13^C_10_,^15^N_5_ ATP	361	78.8	−130	−70	−10

#### Untargeted metabolomics

Primary mouse hepatocytes isolated from Alb-Cre and SPT3-hKO littermate controls were plated overnight and then harvested in ice-cold methanol and homogenized by sonication. The protein precipitate was removed by centrifugation and used for normalization. Metabolomics were carried out on a Thermoscientific Q Exactive HF OrbiTrap with a HILIC column, and data analysis was performed using Compound Discoverer software.

#### U^13^C-glutamine-labeled targeted metabolomics

Cells were serum starved for 3 h in DMEM after which media was aspirated, and cells were rinsed twice with DMEM containing no glucose, serum, glutamine or phenol. Cells were then treated with the same media containing 4 mM U^13^C-Glutamine for 8 h. Cells were harvested in 1 mL ice-cold methanol and then processed according to.^76^ Key metabolites were then assessed by LC-MS/MS according to.^77^

#### Post-treatment with bacterial sphingomyelinase

Primary hepatocytes were post-treated in 6 well dishes. Media was aspirated and replaced with 0.5 mL of 4% PFA, and fixed for 10 min at 37°C. Cells were then washed once with serum-free DMEM, and then treated with 1 mL of serum free DMEM or DMEM containing ≥0.1 units of bacterial sphingomyelinase (bSMase) and reacted for 10 min at 37°C. Media or bSMase was aspirated and then cells were harvested in ice-cold methanol according to the protocol for sphingolipid extraction.

#### Measurement of sphingolipids by LC-ESI-MS/MS

For tissue, 50 mg was homogenized in methanol by magnetic bead homogenizer at the maximum power setting for 3× 3 min cycles, resting in an ice bath for 1 min between cycles. Aliquots were removed diluted 10-fold in PBS for quantification by BCA assay. Protein content was equalized for all samples and then diluted into 2 mL of methanol. For hepatocytes, an equal number of cells isolated from Alb-Cre and SPT3-hKO littermate controls were harvested from 6-well tissue culture dishes. First, they were washed with ice-cold PBS and then harvested in 1 mL of ice-cold methanol and transferred to 13 × 100 mm borosilicate tubes containing 1 mL of ice-cold methanol. Internal standards are purchased from Avanti Polar Lipids (Alabaster, AL). An internal standard cocktail containing 250 pmol of sphingoid bases and sphingoid base 1-phosphates are 17-carbon chain length analogs: C17-sphingosine, (2S,3R,4E)-2-aminoheptadec-4-ene-1,3-diol (d17:1-So); C17-sphinganine, (2S,3R)-2-aminoheptadecane-1,3-diol (d17:0-Sa); C17-sphingosine 1-phosphate, heptadecasphing-4-enine-1-phosphate (d17:1-So1P); and C17-sphinganine 1-phosphate, heptadecasphinganine-1-phosphate (d17:0-Sa1P). Standards for N-acyl sphingolipids are C12-fatty acid analogs: C12-Cer, N-(dodecanoyl)-sphing-4-enine (d18:1/C12:0); C12-Cer1-phosphate, N-(dodecanoyl)-sphing-4-enine-1-phosphate (d18:1/C12:0-Cer1P); C12-sphingomyelin, N-(dodecanoyl)-sphing-4-enine-1-phosphocholine (d18:1/C12:0-SM); and C12-glucosylceramide, N-(dodecanoyl)-1-β-glucosyl-sphing-4-eine was added. Add 1 mL of CHCl3 was added to achieve methanol:chloroform at 2:1. Sphingolipids were measured by LC-ESI-MS/MS with a Sciex 5500 QTRAP (ABSciex, Farmingham, MA) equipped with a Supelco 2.1 (i.d.) × 50 mm Ascentis Express C18 column (Sigma, St. Louis, MO) as previously described.^78^ Following LC-MS/MS analysis total inorganic phosphate content of the final extract was quantified for normalization according to.^79^

Ceramides are readily identified by LC-MS/MS due to fact that first, there are few endogenous metabolites with overlapping mass to charge that coextract with ceramide, which simplifies separation by LC. Second, ceramide readily fragments at moderate collision energies at the sidechain linkage so that the d16 and d18-sphingoid bases illustrated in blue and orange respectively, fragment during tandem MS to reveal the identity of the sidechains illustrated in black and red (Figure 4A). Sphingomyelin however contains a choline group linked by a relatively unstable phosphodiester bond, which is cleaved at low MS/MS collision energies creating a product ion with a mass to charge potentially equal to two or more common ceramide regioisomers. This resulting product ion does not permit resolution of the backbone/sidechain regioisomer combination (Figure 4B). Additionally, taking full advantage of chromatographic separation to aid in accurate sphingomyelin peak identification is complicated by overlapping mass to charge with other abundant and readily ionizable lipid species, especially phosphatidylcholine. For routine measurement of the abundant and easily ionizable d18-based sphingomyelins, these limitations are not a major obstacle especially for samples where non-canonical sphingolipid products can be neglected. However, to identify key SPTLC3-dependent species accurate quantification of the less abundant d16-sphingomyelins is critical. One work-around is a pseudo MS3 approach.^80^ By taking advantage of the ion trapping capability of the Qtrap instrument cleavage products are produced that yield backbone information. However, the limitations of this approach are that first it is not routinely employed, and thus requires additional validation. The second and the major limitation is that there is a substantial loss of sensitivity, and loss of sensitivity is a major obstacle for study of the generally the lower abundance d16-sphingomyelins.

#### Normalization of lipidomics

One-fourth of the Bligh Dyer lipid was placed it in a clean glass tube. Sample were ashed overnight at 160°C in 600 μL of ashing solution alongside standards. Total phosphate was quantified by OD_600_ following addition of 0.9 mL water, 0.5 mL 0.9% ammonium molybdate, 200 μL 9.0% ascorbic acid and then incubating at 45°C for 30 min.

### QUANTIFICATION AND STATISTICAL ANALYSIS

P-values, where indicated, were calculated by a 2-tailed Student’s t-test or ANOVA followed by Dunnett’s multiple comparisons test, where multiple groups were present. Mouse weight data fitted with a linear mixed model with 8 groups defined by combinations of gender, mutation, and diet and interactions of these groups with weeks. P-values for estimated differences in slope were corrected for multiple testing via Tukey correction.

## Supplementary Material

1

## Figures and Tables

**KEY RESOURCES TABLE:** 

REAGENT or RESOURCE	SOURCE	IDENTIFIER

Antibodies		
Phospho-Akt (Ser473) Antibody	Cell Signaling Technology	RRID:AB_329825
Phospho-AMPKα (Thr172)	Cell Signaling Technology	5256
PCK1 (D12F5) Rabbit mAb	Cell Signaling Technology	RRID:AB_2687968
Phospho-CREB (Ser133) (87G3) Rabbit mAb	Cell Signaling Technology	RRID:AB_2561044
Na,K-ATPase α1	Cell Signaling Technology	RRID:AB_2798866

Chemicals, peptides, and recombinant proteins		

Glucagon	Millipore/Sigma	G2044
pCPT-cAMP	Millipore/Sigma	C3912
IBMX	Millipore/Sigma	I5879
Thapsigargin	R&D Biothechne	1138
Digoxin	R&D Biothechne	4583
Sphingomyelinase From Bacillus Cereus	Millipore/Sigma	S9396
Adenosine 5’-Triphosphate	Millipore/Sigma	A-2383
adenosine-^13^ C10,^15^N5, 5′-Triphosphate	Millipore/Sigma	645702
cyclic AMP	MedChemExpress	HY-B1511S
ATP	MedChemExpress	645702
U^13^C-Glutamine	Cambridge Isotopes	CLM-1822

Critical commercial assays		

Liver Glycogen	Abcam	ab65620
Mouse Insulin ELISA kit	Crystal Chem	90080
cAMP ELISA	Cayman	581001

Deposited data		

Raw data can be found at: Montefusco, David, 2024, “SPLTC3 in Fatty Liver Disease”	Harvard Dataverse, V1.	https://doi.org/10.7910/DVN/SMNKQP

Experimental models: Organisms/strains		

Albumin Cre B6.Cg-Speer6-ps1^Tg(Alb-cre)21Mgn^/J	Jackson Laboratories	RRID:IMSR_JAX:003574
SPTLC3 conditional knockout mouse model	Our laboratory	SPTLC3-flox

Oligonucleotides		

PCK1_forward	Eurofins	CTCAGCTGCATA ACGGTCTG
PCK1 reverse	Eurofins	CTTCAGCTTGCG GATGACAC
G6PC forward	Eurofins	TTGCATTCCTGTATGGTAGTGG
G6PC reverse	Eurofins	TAGGCTGAGGAGGAGAAAACTG
GLUT2 forward	Eurofins	AATGGTCGCCTCATTCTTTG
GLUT2 reverse	Eurofins	ATCAAGAGGGCTCCAGTCAA
SNAT2 forward	Eurofins	GCAGTGGAATCCTTGGGCTT
SNAT2 reverse	Eurofins	ATAAAGACCCTCCTTCATTGGCA
SNAT3 forward	Eurofins	TCGGCTACCTGGGTTACTC
SNAT3 reverse	Eurofins	GGGAACAGAACAATCGGAACTG
SNAT4 forward	Eurofins	CCTCGTGCCTACCAT CAAATAC
SNAT4 reverse	Eurofins	AGACCAAAGCCCCAATCTTC
TNFα forward	Eurofins	TTGCTCTGTGAAGGGAAATGG
TNFα reverse	Eurofins	CCTGAGCCATAATCCCCTTTC
MCP1 forward	Eurofins	TTAAAAACCTGGATCGGAACCAA
MCP1 reverse	Eurofins	GCATTAGCTTCAGATTTACGGGT
IL6 forward	Eurofins	TAGTCCTTCCTACCCCAATTTCC
IL6 reverse	Eurofins	TTGGTCCTTAGCCACTCCTTC
IL10 forward	Eurofins	TGAATTCCCTGGGTGAGAAGC
IL10 reverse	Eurofins	CACCTTGGTCTTGGA GCTTATT

## Highlights

- A hepatic SPTLC3 knockout (SPTLC3-hKO) is found to have suppressed hepatic gluconeogenesis
- SPTLC3-hKO is depleted in d16-based sphingomyelin at the plasma membrane (PM)
- SPTLC3 expression is required to sustain wild-type levels of PM adenylate cyclase activity
