# Supplementary material for: SPTLC3 regulates plasma membrane sphingolipid composition to facilitate hepatic gluconeogenesis
Source: Cell Rep. Author manuscript; Available in PMC 2025 Apr 17. (PMC12004358; doi:10.1016/j.celrep.2024.115054)

Cell Reports, Volume *43*

Supplemental information

SPTLC3 regulates plasma membrane sphingolipid composition to facilitate hepatic gluconeogenesis

David Montefusco, Maryam Jamil, Daniel Canals, Siri Saligrama, Yang Yue, Jeremy Allegood, and L. Ashley Cowart

SUPPLEMENTAL FIGURE CAPTIONS

Figure S1. Confirmation of hepatocyte-specific knockout of SPTLC3.

(A) Shematic illustration showing the introduction of flanking loxp sites using a CRISPR Cas9-facilitated homologous recombination of a single stranded DNA megamer (ssMEGAMER). Each loxp sequence includes EcoR1 restriction sites to facilitate routine genotyping. The CRISPR guided sequences (g1/g2) are labeled as well as the location of primers used to confirm excision of exon 8 by the cre recombinase.
(B) Genomic DNA extracted from albumin-cre (WT) and SPT3-hKO primary mouse hepatocytes showing successful excision of exon 8.
(C) For routine genotyping of tail-snip DNA, the PCR amplicon was digested with EcoR1. 420bp: amplified region around exon 8 containing loxp sites, 380bp: amplified region around wild-type exon 8, 234/186bp: EcoR1 digestion production of the 420bp amplicon.

Figure S2. Markers of inflammation were measured on control diet (CD) and high fat diet (HFD) livers by RT-qPCR.

Expression was quantified by ΔΔcq method relative to a reference gene panel consisting of βactin, Hmbs1 and Tbp1. Significance was determined by t-test, **$P<0.01$, N=5.

Figure S3. Female SPT3-hKO do not present a phenotype related to glucose production or steatosis. Female SPT3-hKO and Albumin-Cre controls were placed on high fat diet for 16 weeks.

(A) Blood glucose following a 6 hr fast.
(B) Intraperitoneal glucose tolerance test.
(C) Area under the curve for the glucose tolerance test.
(D) Triglycerides in liver homogenate.
(E) Liver weight.
(F) H&E staining, scale bars are 200 µm.

All graphical data are represented as mean ± SEM. P-values calculated by t-test,*$P<0.05$,**$P<0.01$,***$P<0.005$, N=5.

Figure S4. Untargeted metabolomics of primary mouse hepatocytes.

Average of N=5 replicates. Red and green metabolites higher or lower in SPT3-hKO (KO) versus Alb-Cre (WT) respectively and are selected as significant based on an adjusted P-value of less than 0.05.

Figure S5. Lipidomics of d18.1-Ceramides Following bSMase Post-Treatment.

D18.1-based ceramide levels determined by LC-MS/MS on Alb-Cre and SPT3-hKO primary mouse hepatocytes with control or bacterial sphingomyelinase (bSMase) post-treatment. P-values determined by ANOVA, **$P<0.05$,****$P<0.0001$. N=5 biological replicates.

Figure S6. Expression of gluconeogenic genes in post-prandial mouse livers.

Expression in liver tissue homogenate of mice following high fat diet (HFD), versus low-glycemic control diet (CD). Expression was quantified by ΔΔcq method with a reference gene panel consisting of βactin, Tbp1, and Hmbs1. Data are represented as mean ± SEM. P-values calculated by t-test. N=5 biological replicates.

Figure S7: Uncropped version of PCK1 and Vinculin loading control for Alb-Cre and SPTLC3-hKO primary mouse hepatocytes treated with glucagon that appears in Figure 2C.

Figure S8: Uncropped version of P-CREB/CREB and Vinculin loading control for Alb-Cre and SPTLC3-hKO primary mouse hepatocytes treated with glucagon that appears in Figure 2D.

Figure S9: Uncropped version of Na K ATPase Western blot for isolated plasma membrane fractions that appear in Figure 3C.

Figure S1. Confirmation of hepatocyte-specific knockout of SPTLC3.

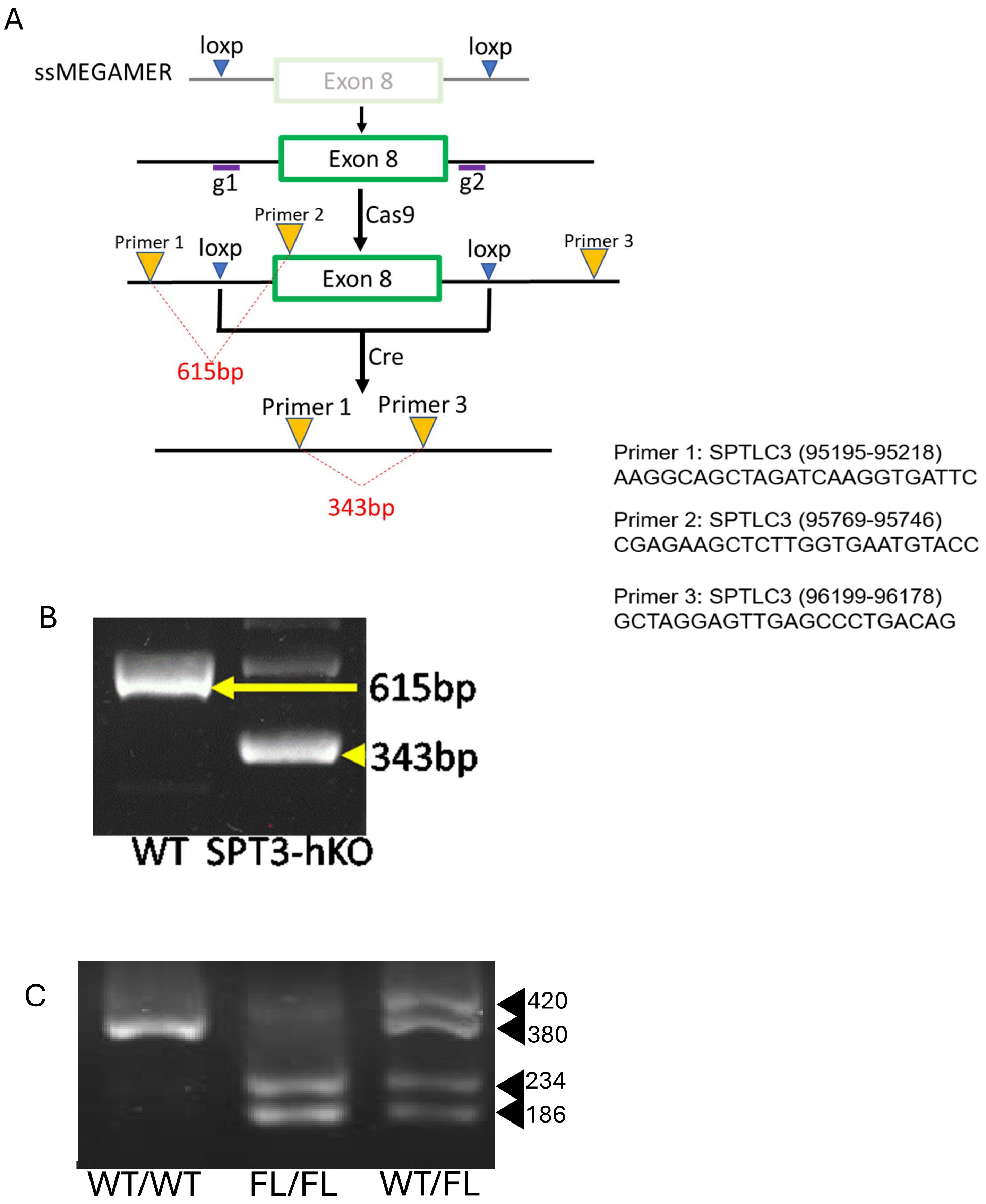

Figure S2. Markers of inflammation were measured on control diet (CD) and high fat diet (HFD) livers by RT-qPCR.

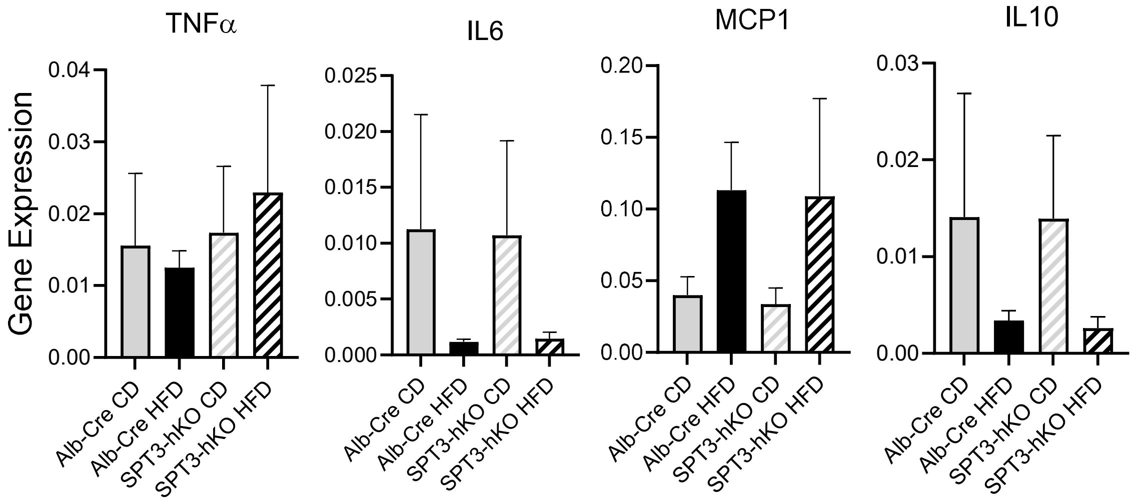

Figure S3. Female SPT3-hKO do not present a phenotype related to glucose production or steatosis. Female SPT3-hKO and Albumin-Cre controls were placed on high fat diet for 16 weeks.

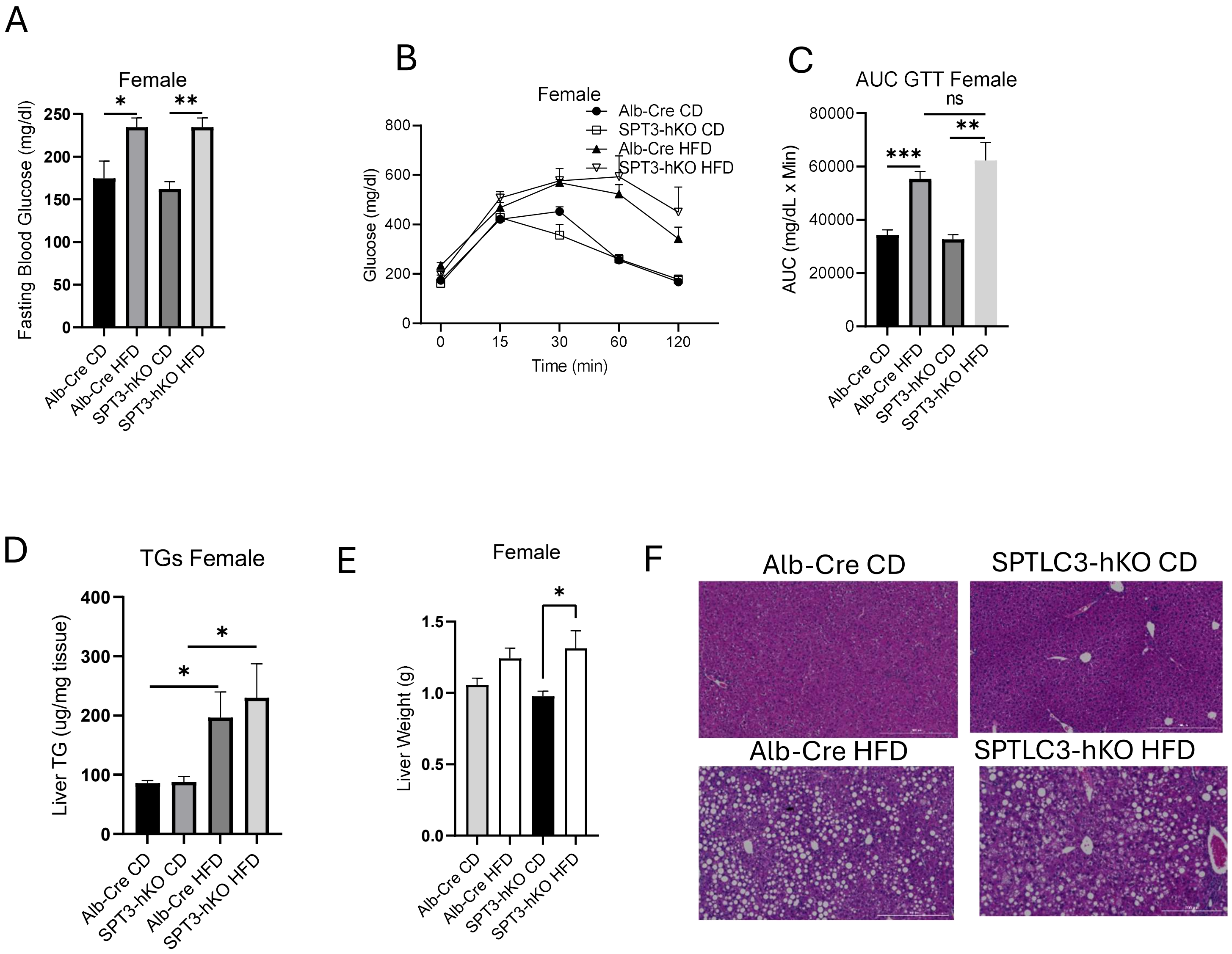

Figure S4. Untargeted metabolomics of primary mouse hepatocytes.

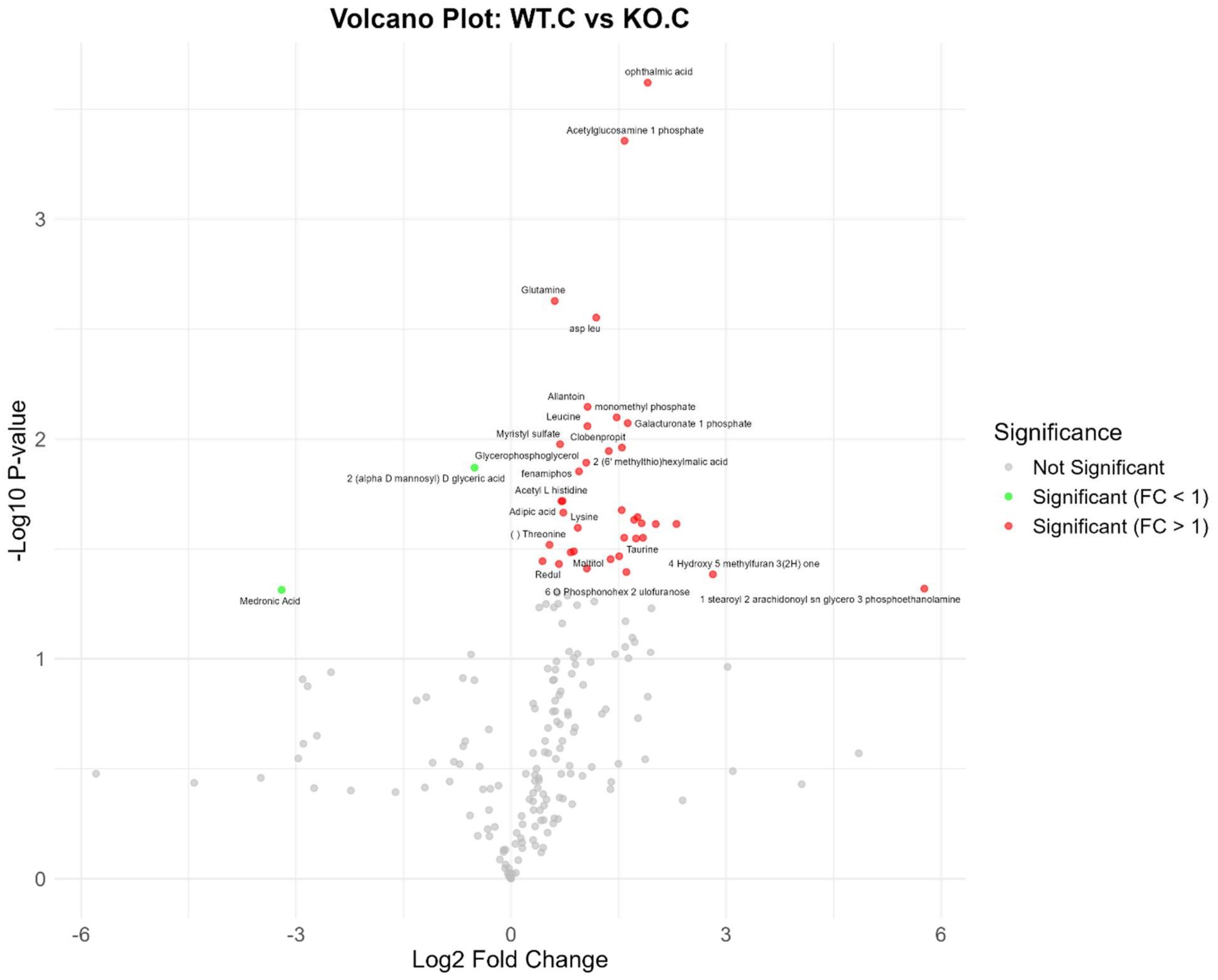

Figure S5. Lipidomics of d18.1-Ceramides Following bSMase Post-Treatment.

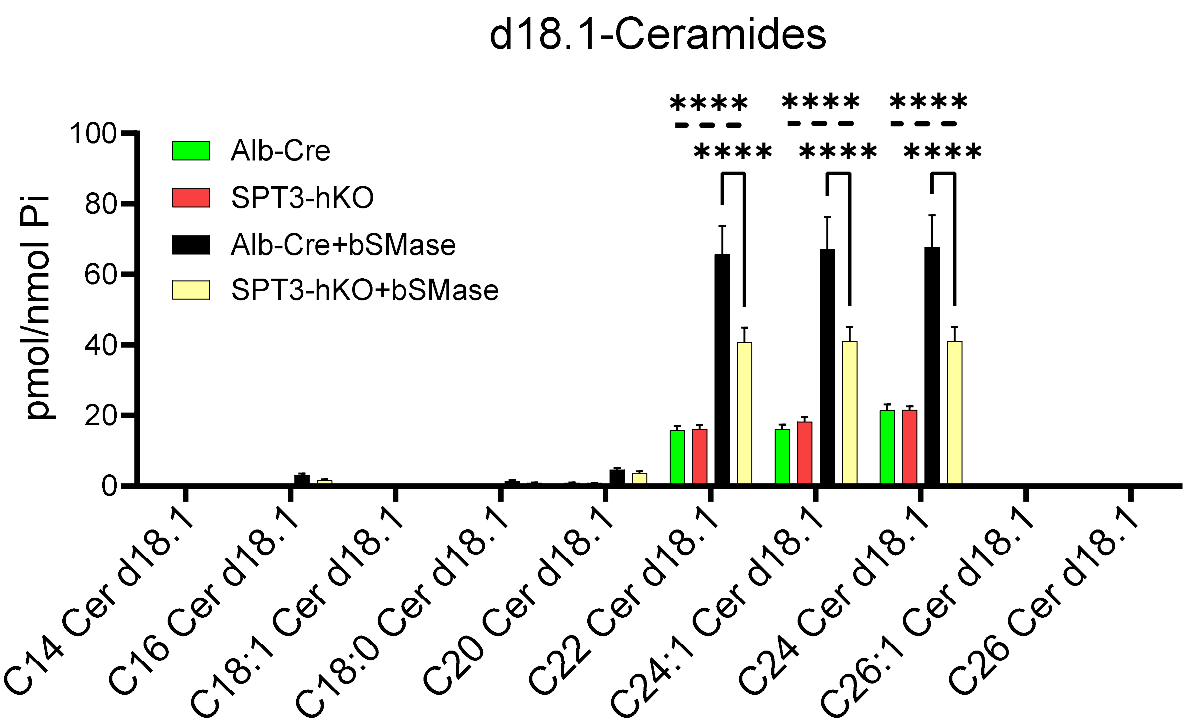

Figure S6. Expression of gluconeogenic genes in post-prandial mouse livers.

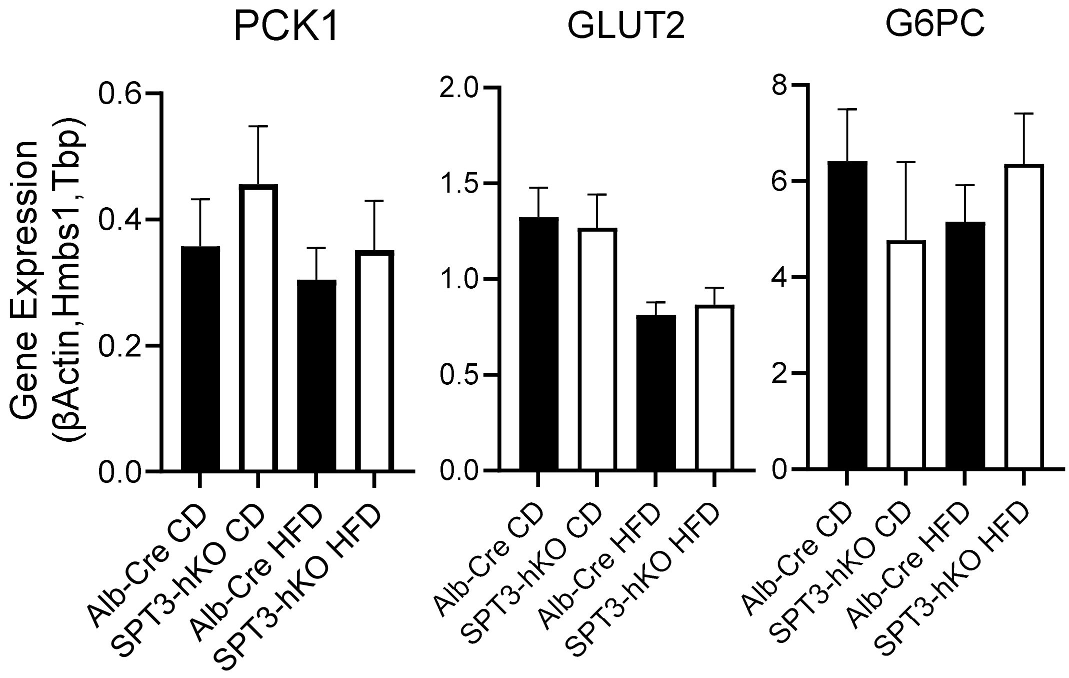

Figure S7: Uncropped version of PCK1 and Vinculin loading control for Alb-Cre and SPTLC3-hKO primary mouse hepatocytes treated with glucagon that appears in Figure 2C.

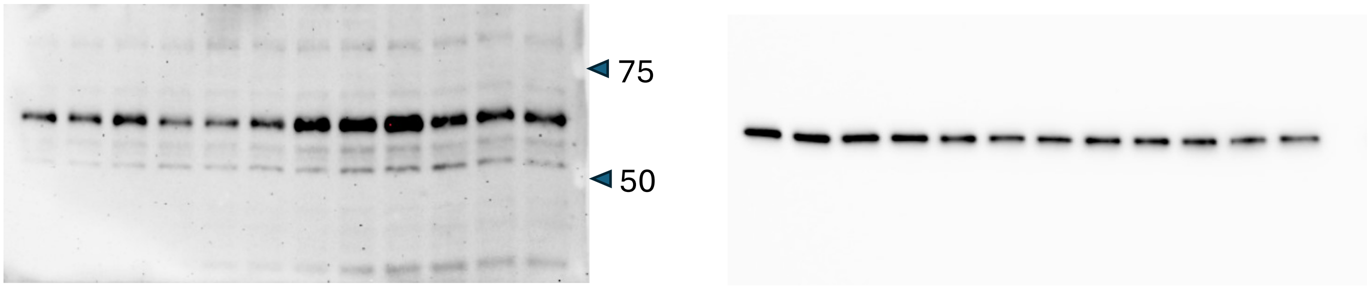

Figure S8: Uncropped version of P-CREB/CREB and Vinculin loading control for Alb-Cre and SPTLC3-hKO primary mouse hepatocytes treated with glucagon that appears in Figure 2D.

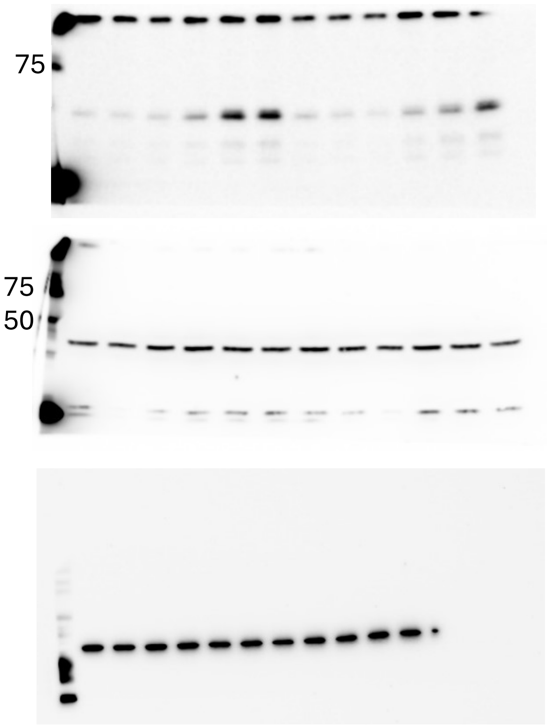

Figure S9: Uncropped version of Na K ATPase Western blot for isolated plasma membrane fractions that appear in Figure 3C.

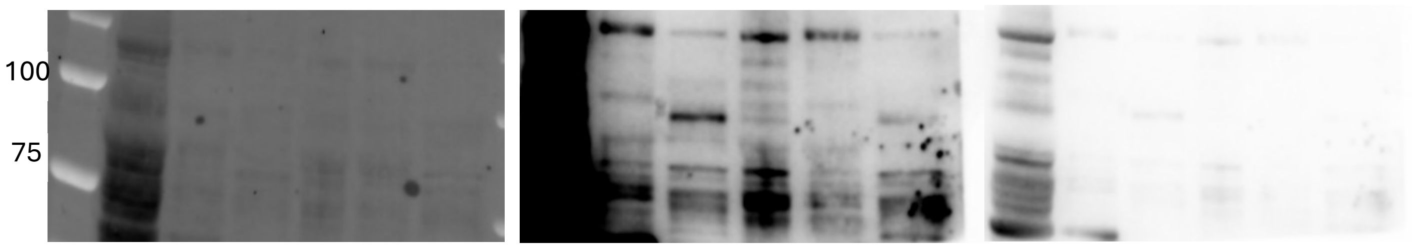

Supplement: 1 [file NIHMS2061147-supplement-1.pdf]
